# Measures of Adiposity and Cardiovascular Disease Risk Factors, New York City Health and Nutrition Examination Survey, 2004

**Published:** 2011-04-15

**Authors:** R. Charon Gwynn, Magdalena Berger, Elizabeth Needham Waddell, Lorna E. Thorpe, Renu K. Garg, Robyn Philburn

**Affiliations:** Mailman School of Public Health, Columbia University; New York City Department of Health and Mental Hygiene, New York, New York; New York City Department of Health and Mental Hygiene, New York, New York; New York City Department of Health and Mental Hygiene, New York, New York; University of Washington, Seattle, Washington; Forest Research Institute, New York, New York

## Abstract

**Introduction:**

Body mass index (BMI) and indicators of central adiposity have been associated with cardiovascular disease (CVD) risk factors, but ambiguity remains about which measure optimally predicts CVD risk and is best suited for different racial/ethnic groups. We sought to characterize excess adiposity among New York City adults and assess the potential associations between multiple adiposity indicators and CVD risk factors, by race/ethnicity.

**Methods:**

The New York City Health and Nutrition Examination Survey (NYC HANES) is a population-based survey of noninstitutionalized New York City adult residents aged 20 years or older. We compared the prevalence of obesity (BMI ≥30 kg/m^2^), elevated waist circumference (>102 cm for men, >88 cm for women), and elevated waist-to-height ratio (≥0.5) for participants in the 2004 NYC HANES (n = 1,912) and the 2003-2004 National Health and Nutrition Examination Survey (n = 4,075). Logistic regression was used to assess potential associations between each of these indicators of excess adiposity and CVD risk factors (diabetes, impaired fasting glucose, hypertension, and hypercholesterolemia), overall and by race/ethnicity.

**Results:**

The prevalence of obesity among NYC HANES participants was 26% and of elevated waist circumference was 46%, both significantly lower than national estimates (31% and 52%, respectively), whereas the prevalence of elevated waist-to-height ratio was higher (82% vs 79%). Most measures of excess adiposity were significantly associated with all CVD risk factors. No single measure of excess adiposity emerged as most consistently predictive of CVD risk in the general population or by race/ethnicity.

**Conclusion:**

New York City has a lower prevalence of obesity and elevated waist circumference but a higher prevalence of elevated waist-to-height ratio than found nationally. Further investigation into the optimal adiposity measure to predict CVD risk across racial/ethnic populations may be warranted.

## Introduction

Obesity, a body mass index (BMI) of at least 30 kg/m^2^, is a standard measure of excess adiposity used to assess health risk nationally and internationally. The prevalence of obesity has risen from 23% to 32% among US adults in the past decade (1) and has been linked to increases in cardiovascular disease (CVD) risk factors (2) as well as death rates from CVD and all causes (3).

Although BMI is a widely accepted measure of overall body mass, some findings suggest that measures of central adiposity have stronger associations with CVD. Two Australian studies found that waist-to-hip ratio was more strongly correlated with CVD risk than was BMI, waist circumference, or waist-to-height ratio (4,5). Alternatively, recent work indicated that waist-to-height ratio was more strongly associated with diabetes, hypertension, and dyslipidemia than were other adiposity measures (6). Other studies maintain that BMI, or BMI in conjunction with measures of central adiposity, predicts CVD outcomes as well as or better than central adiposity measures (7). Recent findings have not consistently shown a single indicator to be more highly associated with CVD risk, but the relationship is further complicated by the varying associations between adiposity measures and CVD risk by race/ethnicity (8). Ambiguity remains about which adiposity measure optimally predicts CVD risk and is best suited for different racial/ethnic groups.

New York City is a diverse urban community, with a higher percentage of black, Hispanic, and low-income residents — all groups with documented high prevalence of obesity — than national averages (1). Although recent studies have quantified racial/ethnic disparities in CVD risk factors in New York City (9,10), none has assessed the racial/ethnic differences in associations between multiple adiposity measures and CVD risk factors.

We used data from the 2004 New York City Health and Nutrition Examination Survey (NYC HANES) to characterize excess adiposity (obesity, elevated waist circumference, and elevated waist-to-height ratio) among New York City adults. We then compared local estimates to similarly measured national estimates by using National Health and Nutrition Examination Survey (NHANES) 2003-2004 data. We further assessed the association between each adiposity measure and CVD risk factors, overall and by race/ethnicity.

## Methods

NYC HANES is a population-based, cross-sectional survey of noninstitutionalized New York City adult residents. Modeled after NHANES, the 2004 NYC HANES used a 3-stage probability design to select a representative sample of participants aged 20 years or older. The survey included a physical examination, laboratory testing, and a face-to-face and computer-assisted self-interview. A detailed description of the study's methods has been published elsewhere (11).

All measurements and specimen collection were conducted by using standardized protocols and equipment consistent with those used for NHANES (12). We excluded pregnant women (n = 29) and participants without valid anthropometric values (n = 58), for a final sample of 1,912 participants. For analyses of diabetes, fasting blood glucose was measured from a subsample of participants who fasted for 8 hours (n = 1,275). A detailed description of the fasting sample is provided elsewhere (10).

NHANES is an ongoing population-based, cross-sectional survey of noninstitutionalized US residents aged 2 months or older (12). Similar to NYC HANES, NHANES uses a multistage cluster sampling design. To be consistent with NYC HANES, we analyzed NHANES 2003-2004 data for adults aged 20 years or older and excluded pregnant women and participants without valid anthropometric values, for a final sample of 4,075 participants.

Participants with a BMI of at least 30 kg/m^2^ were classified as obese (13). We defined elevated waist circumference as greater than 102 cm in men and greater than 88 cm in women (13) to be consistent with cutoffs developed by the National Institutes of Health to identify the risk for obesity-related health outcomes in overweight and obese adults. We defined waist-to-height ratio as waist circumference in centimeters divided by height in centimeters. Because no established cutoffs for waist-to-height ratio exist, we used a value of 0.5 or more, proposed by Ashwell and Hsieh, as being indicative of increased health risk across sex and race/ethnicity groups (14).

Lipid profiles were analyzed at the Lipoprotein Analytical Laboratory at Johns Hopkins University Hospital. Blood glucose levels were measured at the University of Missouri Diabetes Diagnostic Laboratory according to NHANES protocols (15).

We defined hypertension as a systolic blood pressure measurement of at least 140 mm Hg or a diastolic blood pressure measurement of at least 90 mm Hg or currently taking prescribed antihypertensive medications. We defined hypercholesterolemia as a total cholesterol measurement of at least 240 mg/dL or currently taking prescribed cholesterol-lowering medications (16). We defined diabetes as a fasting plasma glucose measurement of at least 126 mg/dL or participant self-report that a health care provider had ever told them that they had diabetes (other than during pregnancy for women). Impaired fasting glucose (IFG) was defined as having a fasting plasma glucose measurement of 100 to 125 mg/dL among participants who did not self-report diabetes (17).

Demographic variables were age, sex, race/ethnicity, education, income, nativity, smoking, exercise, and alcohol use. We defined nativity as US-born or foreign-born (participants born in Puerto Rico, US Virgin Islands, any other US territory, or anywhere else other than the 50 states and Washington, DC). We defined exercise as vigorous activity (≥20 min on ≥3 d/wk), some activity, or no activity (18).

We categorized respondents into smokers and nonsmokers. Smokers were defined as respondents who reported smoking at least 100 cigarettes in their life and answered "every day" or "some days" to the question, "Do you now smoke cigarettes?" Nonsmokers answered no to the question, "Have you smoked ≥100 cigarettes in your entire life?" or to the question "Do you now smoke cigarettes?" We defined alcohol use as heavy (≥2 drinks/d for men, ≥1 drink/d for women), moderate (<2 drinks/d but >12 drinks/y for men, <1 drink/d but >12 drinks/y for women), and low/none (those who had never had a drink or <12 drinks/y).

To account for differential selection probabilities and survey nonresponse we weighted participant data; weights were poststratified to reflect population totals. We further adjusted weights for item nonresponse on adiposity indicators and CVD outcomes based on age group, race/ethnicity, and sex. We conducted statistical analysis using SAS version 9.0 (SAS Institute, Inc, Cary, North Carolina) and used SUDAAN version 10.0 (RTI International, Research Triangle Park, North Carolina) to obtain standard error estimates and 95% confidence intervals (CIs) by Taylor series linearization. We age-adjusted New York City and national prevalence estimates to the 2000 US standard population aged 20 years or older (19). Significance was defined as *P* < .05. We calculated relative standard errors for all means and percentages; estimates with relative standard errors of more than 30% are considered unreliable. To test differences between NYC HANES and NHANES survey estimates, we used a linear contrast *t* test.

To assess associations between each adiposity measure and the hypertension and hypercholesterolemia outcomes we used logistic regression. For association between each adiposity measure and a 3-level indicator of glucose metabolism (diabetes, IFG, and normal glucose metabolism), we used multinomial regression. Base models included the CVD risk factor as the dependent variable and known confounders (age, sex, race/ethnicity, education, income, nativity, exercise, smoking, and alcohol use) as the independent variables. We included each adiposity measure in a separate base model for each of 3 CVD outcomes. Models were also stratified by race/ethnicity.

## Results

### NYC HANES versus NHANES

The overall age-adjusted prevalence of obesity among New York City adults (26%) was significantly lower than the national prevalence (31%) (Table 1). The prevalence of obesity among New York City middle-aged adults (*P* = .02) and adults who were young, men, white, black, had at least a high school education, or had an annual household income of at least $20,000 (*P* < .01) was significantly lower than that of their national counterparts. The overall prevalence of elevated waist circumference among New York City adults was 46%, also significantly lower than the national prevalence of 52% (*P* < .01).

The prevalence of elevated waist-to-height ratio was significantly higher among New York City adults than among US adults (82% vs 79%, *P* = .03). Specifically, the prevalence was significantly higher among New York City adults aged 60 or older, women, those with at least a high school education, or those with an annual household income of less than $20,000 compared with adults nationwide (*P* < .05). Direct comparisons between New York City's Hispanic population and that from NHANES are not possible because of differing regions of origin. However, both groups had a similarly high prevalence of elevated waist-to-height ratio (90% for New York City Hispanics vs 89% for US Mexican Americans and 83% for other US Hispanics).

### Excess adiposity and CVD risk

**Diabetes and IFG**

After adjusting for covariates, we found that people with excess adiposity for any measure had approximately 3 times the odds of having diabetes as those with normal adiposity (Table 2). Overall, obese participants and those with elevated waist-to-height ratio were twice as likely to have IFG. Among whites, both obesity and elevated waist circumference had large associations with diabetes (Table 3); however, estimates had wide CIs. Among Hispanics, obesity was associated with diabetes and among whites it was associated with IFG. Elevated waist-to-height ratio was significantly associated with IFG among Hispanics; however, CIs were wide.

**Hypertension**

Overall, obese participants and those with elevated waist-to-height ratio had more than 2.5 times the odds of having hypertension as either nonobese participants or those with normal waist-to-height ratios. We found a smaller but significant association among those with elevated waist circumference. We found significant associations for obesity and hypertension for all racial/ethnic groups. Elevated waist-to-height ratio was significantly associated with hypertension among whites and Hispanics; however, CIs were wide.

**Hypercholesterolemia**

All adiposity measures had similar associations with hypercholesterolemia after adjusting for covariates. Participants with elevated adiposity were about twice as likely to have hypercholesterolemia as those with normal measures. Whites had significantly higher odds of hypercholesterolemia for obesity, elevated waist circumference, and elevated waist-to-height ratio. Hispanics with elevated waist-to-height ratio were at significantly higher risk for hypercholesterolemia, but the CI was wide. Among Asians, obesity was significantly associated with hypercholesterolemia.

## Discussion

We observed a lower prevalence of obesity and elevated waist circumference in New York City than nationally but a higher prevalence of elevated waist-to-height ratio. In the New York City adult population, most measures of excess adiposity were significantly associated with increased odds of diabetes, IFG, hypertension, and hypercholesterolemia. No single adiposity indicator was consistently associated with the CVD risk factors across all racial/ethnic groups, but obesity was consistently associated with hypertension across all racial/ethnic groups.

The lower prevalence of obesity in New York City compared with the United States is notable, given that New York City has a larger proportion of blacks, Hispanics, and people of low socioeconomic status (20,21), groups that typically have a higher prevalence of obesity (22). Our study confirms higher obesity prevalence in blacks, Hispanics, and people of lower socioeconomic status compared with others in New York City, but we found that the prevalence of obesity and elevated waist circumference in New York City subgroups are both lower than found nationally. Lower obesity may result from New York City's urban setting; adults in the New York metropolitan area have lower rates than other US adults of car use and higher rates of active commuting (walking or bicycling to work) (23,24), which have been linked to increased physical activity and lower prevalence of excess adiposity (25). New York City also has a large immigrant population (nearly 40% of the New York City adult population is foreign-born), who have a lower prevalence of obesity than US-born New Yorkers.

Despite the lower prevalence of obesity and elevated waist circumference, we found significantly higher prevalence of elevated waist-to-height ratio in New York City than in the United States. This finding may be the result of the large New York City Hispanic population and its substantially higher waist-to-height ratios. Higher waist-to-height values among Hispanics have been documented in at least 1 other study, which showed that Mexicans residing in Mexico City, Mexican Americans residing in San Antonio, Texas, and white residents from Spanish towns had higher mean waist-to-height ratios compared with non-Hispanic whites in San Antonio (26). However, little is known about waist-to-height ratio in Hispanic groups other than Mexicans. New York City Hispanics are primarily of Puerto Rican and Dominican descent (20), and, based on these findings, appear to also have higher waist-to-height ratio than other racial/ethnic groups.

Several studies (4-6) support our results that most adiposity measures were significantly associated with increased odds of diabetes, IFG, hypertension, and hypercholesterolemia. However, in race/ethnicity-specific analyses, these results were less consistent. Obesity was associated with hypertension across all racial/ethnic groups, but obesity and other adiposity indicators were not consistently associated with other CVD outcomes (eg, among whites and Hispanics, elevated waist-to-height ratio was associated with both hypertension and hypercholesterolemia, but among blacks it was associated with diabetes).

Previous findings have demonstrated that associations between adiposity measures and CVD risk differ by race/ethnicity. Okosun et al found that excess abdominal adiposity (as measured by elevated waist circumference) was associated with an increased risk of prehypertension, hypertension, and multiple metabolic syndrome (defined as 2 or more of the following: hypertension, diabetes, dyslipidemia, hyperinsulinemia, or hypertriglyceridemia) in whites, blacks, and Hispanics; the associations often were strongest for blacks and Hispanics (27-29). Furthermore, Ghandehari et al found that both BMI and waist circumference were associated with mean levels of fasting glucose, blood pressure, and cholesterol among whites, blacks, and Hispanics; however, this relationship was attenuated among blacks compared with other racial/ethnic groups (30). In this study, obesity was consistently associated with hypertension across the racial/ethnic groups, but we did not find consistent associations of elevated waist circumference and obesity with all of the examined CVD risk factors. Differing findings may be due to the small sample size in our analysis, thereby limiting statistical power to observe significant associations.

Our analyses are based on the use of accepted or proposed cutoff values for each adiposity indicator. Other studies have assessed adiposity indicators as linear predictors or quartiles of linear predictors or have identified cutoffs based on receiver operating curves (6,31,32). For example, Janssen and coauthors concluded that waist circumference was a better predictor of health endpoints than was BMI when waist circumference was evaluated as a continuous variable (33). Our objective was not to identify optimal values for predicting CVD risk factors, but we recognize that the explanatory power of the adiposity indicators is limited by the predefined cutoff values used. Furthermore, because considerations such as ease of use, interpretability, cost, and feasibility are important criteria for acceptability (34), we thought that an assessment based on accepted cutoffs would offer the most practical application.

An additional limitation is the small sample sizes used in some subgroup assessments, which resulted in large CIs, reducing power to assess significant differences in associations. Furthermore, NYC HANES is a cross-sectional survey, and causal inferences regarding the observed associations cannot be made. Finally, data are subject to potential sources of error, including recall bias for self-reported information and measurement error in the examination components. However, standardized quality assurance procedures from NHANES protocols were in place to limit measurement error.

In summary, the prevalence of excess adiposity as measured by obesity and elevated waist circumference in New York City was lower than national rates. New York City had higher prevalence of elevated waist-to-height ratio compared with the United States, likely because of New York City's large Hispanic population. Furthermore, our results show that in the general population, all 3 measures of excess adiposity were significantly associated with CVD risk factors; however, race/ethnicity-specific results were less consistent. Further investigation into the optimal adiposity measure to predict CVD risk across racial/ethnic populations may be warranted.

## Figures and Tables

**Table 1:** Prevalence of Indicators of Excess Adiposity, by Selected Demographic Characteristics, NYC HANES, 2004, and NHANES, 2003-2004

Characteristic	NYC HANES				NHANES			

	n^a^	Obese,^b^ % (95% CI)	Elevated WC,^b^ % (95% CI)	Elevated WHR,^b^ % (95% CI)	n^a^	Obese,^b^ % (95% CI)	Elevated WC,^b^ % (95% CI)	Elevated WHR,^b^ % (95% CI)
**Total**	1,912	25.6 (23.1-28.3)	46.2 (43.5-48.9)	81.9 (79.9-83.7)	4,075	31.2 (28.5-33.9)^c^	51.9 (49.4-54.5)^c^	78.8 (76.5-80.9)^c^
**Age, y^d^**								
20-39	938	20.2 (17.2-23.6)	31.5 (27.9-35.3)	70.2 (66.7-73.6)	1,368	27.8 (24.5-31.4)^c^	37.8 (34.3-41.3)^c^	65.6 (62.2-68.9)
40-59	727	29.5 (25.9-33.5)	52.8 (48.8-56.8)	84.9 (81.8-87.6)	1,261	35.9 (32.0-39.9)^c^	57.8 (54.5-61.0)^c^	84.7 (81.0-87.8)
≥60	247	28.6 (23.0-34.8)	60.8 (54.6-66.7)	96.9 (94.1-98.4)	1,446	29.4 (26.5-32.4)	66.8 (62.8-70.6)	91.8 (89.4-93.8)^c^
**Sex**								
Male	809	22.1 (19.0-25.6)	25.9 (22.6-29.5)	78.0 (74.7-80.9)	2,059	30.0 (27.0-33.1)^c^	42.4 (39.9-44.9)^c^	80.5 (78.0-82.9)
Female	1,103	28.8 (25.4-32.4)	63.7 (60.1-67.2)	85.1 (83.0-87.0)	2,016	32.3 (28.7-36.1)	61.0 (57.1-64.8)	77.2 (73.7-80.3)^c^
**Race/ethnicity**								
Non-Hispanic white	578	21.9 (18.2-26.0)	42.8 (38.4-47.3)	79.8 (76.5-82.8)	2,159	29.8 (27.0-32.8)^c^	51.7 (48.0-55.3)^c^	77.3 (74.2-80.0)
Non-Hispanic black	413	32.1 (26.8-38.0)	51.7 (47.0-56.3)	79.3 (75.6-82.5)	829	43.9 (39.4-48.5)^c^	57.6 (54.7-60.4)^c^	80.0 (76.8-82.8)
Non-Hispanic Asian	253	6.6 (4.0-10.7)	26.7 (20.1-34.4)	74.1 (67.8-79.5)	NA	NA	NA	NA
Hispanic	638	32.5 (28.2-37.2)	54.6 (50.2-59.0)	89.9 (88.0-91.5)	NA	NA	NA	NA
Non-Hispanic other	27	21.1 (10.3-38.4)^e^	33.9 (16.3-57.4)^e^	79.4 (67.4-87.8)^e^	141	13.7 (8.5-21.3)	33.7 (24.3-44.6)	73.3 (62.3-82.0)
Mexican American	NA	NA	NA	NA	816	36.4 (32.1-41.0)	54.8 (49.3-60.2)	89.4 (86.1-91.9)
Other Hispanic	NA	NA	NA	NA	130	28.6 (19.3-40.3)	50.8 (42.6-58.9)	82.8 (77.0-87.4)
**Education**								
<High school	540	33.2 (28.5-38.2)	52.7 (47.7-57.7)	85.8 (82.0-88.9)	2,199	33.5 (31.0-36.2)	54.3 (51.5-57.0)	82.7 (79.0-85.8)
≥High school	1,365	22.7 (19.9-25.7)	43.7 (40.7-46.7)	80.6 (78.2-82.7)	1,872	29.4 (26.0-33.0)^c^	50.0 (46.2-53.8)^c^	76.0 (73.3-78.5)^c^
**Annual household income, $**								
<20,000	616	27.3(23.5-31.4)	47.0 (42.4-51.7)	84.0 (81.0-86.7)	1,269	30.6 (27.1-34.3)	52.2 (49.2-55.2)	77.2 (73.6-80.4)^c^
≥20,000	1,239	25.2 (22.0-28.6)	45.6 (42.2-49.0)	81.2 (78.9-83.3)	2,689	31.1 (27.9-34.5)^c^	51.9 (48.8-55.0)^c^	79.5 (77.0-81.9)
**Nativity**								
US-born	851	30.0 (26.1-34.1)	48.6 (44.6-52.6)	81.2 (78.7-83.5)	3,220	32.7 (29.9-35.6)	53.7 (51.1-56.4)	78.7 (76.3-80.9)
Foreign-born^f^	1,055	21.9 (18.9-25.1)	44.0 (40.6-47.5)	82.5 (79.9-84.8)	854	22.9 (18.8-27.6)	41.9 (37.1-47.0)	79.0 (75.6-82.1)

Abbreviations: NYC HANES, New York City Health and Nutrition Examination Survey; NHANES, National Health and Nutrition Examination Survey; CI, confidence interval; WC, waist circumference; WHR, waist-to-height ratio; NA, not assessed.

a Total may not equal the sum of subgroups because of missing data.

b Obesity defined as ≥30 kg/m^2^. Elevated waist circumference defined as >102 cm for men, >88 cm for women. Elevated waist-to-height ratio defined as ≥0.5.

c Significant difference between NYC HANES and NHANES at *P* < .05.

d Estimates are not age-adjusted.

e The relative standard error for this estimate was >30% or the denominator was <50, indicating that it may be unstable and should be interpreted with caution.

f Participants born in Puerto Rico, US Virgin Islands, any other US territory, or anywhere other than the 50 states and Washington, DC.

**Table 2:** Odds of Associations Between Indicators of Excess Adiposity and Risk Factors for Cardiovascular Disease, NYC HANES, 2004

**Indicator^a^**	OR^b^ (95% CI)	AOR^c^ (95% CI)
**Diabetes,^d^ n = 1,253**		
Obese	3.2 (2.0-5.2)	3.5 (2.0-6.3)
Elevated waist circumference	3.0 (1.9-4.8)	3.3 (1.8-6.0)
Elevated waist-to-height ratio	5.2 (2.4-11.6)	3.0 (1.2-7.2)
**Impaired fasting glucose,^d^ n = 1,253**		
Obese	2.1 (1.5-3.0)	2.4 (1.6-3.5)
Elevated waist circumference	1.3 (1.0-1.9)	1.5 (1.0-2.3)
Elevated waist-to-height ratio	2.7 (1.7-4.2)	2.1 (1.3-3.4)
**Hypertension,^e^ n = 1,811**		
Obese	2.4 (1.9-3.0)	2.7 (1.9-3.6)
Elevated waist circumference	2.3 (1.8-3.0)	1.5 (1.1-2.2)
Elevated waist-to-height ratio	5.9 (3.6-9.7)	2.6 (1.5-4.4)
**Hypercholesterolemia,^f^ n = 1,646**		
Obese	1.8 (1.3-2.4)	1.9 (1.3-2.6)
Elevated waist circumference	2.2 (1.7-2.9)	1.9 (1.3-2.9)
Elevated waist-to-height ratio	3.3 (2.2-4.8)	2.3 (1.5-3.5)

Abbreviations: NYC HANES, New York City Health and Nutrition Examination Survey; OR, odds ratio; CI, confidence interval; AOR, adjusted odds ratio.

a Obesity defined as body mass index ≥30.0 kg/m^2^ (reference <30 kg/m^2^). Elevated waist circumference defined as >102 cm for men (reference ≤102 cm) and >88 cm for women (reference ≤88 cm). Elevated waist-to-height ratio defined as ≥0.5 (reference <0.5).

b Not adjusted for covariates in base model.

c Adjusted for covariates in base model.

d Results from a multinomial model with 3 levels (diabetes, impaired fasting glucose, normal). Base model accounts for age, sex, race/ethnicity, education, income, exercise, and nativity.

e Base model accounts for age, sex, race/ethnicity, education, income, exercise, nativity, smoking, and alcohol use.

f Base model accounts for age, sex, race/ethnicity, education, income, exercise, nativity, and smoking.

**Table 3:** Odds of Associations Between Indicators of Excess Adiposity and Risk Factors for Cardiovascular Disease, by Race/Ethnicity, NYC HANES, 2004

Indicator^a^	Race/Ethnicity							

	Non-Hispanic White		Non-Hispanic Black		Asian		Hispanic	

	n	AOR (95% CI)	n	AOR (95% CI)	n	AOR (95% CI)	n	AOR (95% CI)
**Diabetes^b^**								
Obese	363	17.3 (5.0-59.2)	279	1.1 (0.5-2.7)	152	2.3 (0.3-17.1)	459	2.4 (1.1-5.5)
Elevated waist circumference	363	7.3 (2.4-22.7)	279	1.7 (0.7-4.4)	152	3.6 (0.5-25.7)	459	2.2 (0.7-6.6)
Elevated waist-to-height ratio	363	3.5 (0.4-38.7)	279	3.1^c^ (1.0-9.1)	NA^d^	NC	467	0.8 (0.2-2.9)
**Impaired fasting glucose^b^**								
Obese	363	5.2 (2.5-10.9)	279	1.4 (0.7-2.7)	152	1.5 (0.3-7.4)	459	1.9 (0.9-3.8)
Elevated waist circumference	363	1.5 (0.7-3.1)	279	1.8 (0.7-4.3)	152	2.2 (0.7-7.2)	459	1.5 (0.8-2.9)
Elevated waist-to-height ratio	363	1.8 (0.8-4.0)	279	3.0 (0.9-9.6)	NA^d^	NC	459	9.0 (2.6-31.7)
**Hypertension^e^**								
Obese	562	2.4 (1.3-4.6)	396	2.7 (1.6-4.5)	238	3.5 (1.1-11.2)	615	3.2 (1.7-5.9)
Elevated waist circumference	562	1.9 (1.0-3.6)	396	1.7 (0.8-3.6)	238	1.0 (0.4-2.5)	615	1.4 (0.8-2.5)
Elevated waist-to-height ratio	562	3.1 (1.1-9.0)	396	2.3 (1.0-5.5)	238	1.2 (0.5-3.3)	615	5.7 (1.4-23.4)
**Hypercholesterolemia^f^**								
Obese	488	2.0 (1.1-3.5)	357	2.1 (1.0-4.4)	212	5.3 (1.2-23.7)	589	1.7 (0.9-2.9)
Elevated waist circumference	488	2.3 (1.2-4.3)	357	2.3 (0.8-6.7)	212	2.0 (0.9-4.8)	589	1.6 (0.9-2.8)
Elevated waist-to-height ratio	488	2.8 (1.4-5.9)	357	1.4 (0.5-3.7)	212	2.0 (0.8-5.2)	589	5.6 (1.9-16.3)

Abbreviations: NYC HANES, New York City Health and Nutrition Examination Survey; AOR, adjusted odds ratio; CI, confidence interval; NA, not applicable; NC, not calculated.

a Obesity defined as body mass index ≥30.0 kg/m^2^ (reference <30 kg/m^2^). Elevated waist circumference defined as >102 cm for men (reference ≤102 cm) and >88 cm for women (reference ≤88 cm). Elevated waist-to-height ratio defined as ≥0.5 (reference <0.5).

b Results from a multinomial model with 3 levels (diabetes, impaired fasting glucose, normal). Base model accounts for age, sex, race/ethnicity, education, income, exercise, and nativity.

c Odds are significant at *P* =.04; the CI includes 1 because of rounding.

d Value could not be calculated because no Asians with a normal waist-to-height ratio had diabetes or impaired fasting glucose.

e Base model accounts for age, sex, race/ethnicity, education, income, exercise, nativity, smoking, and alcohol use.

f Base model accounts for age, sex, race/ethnicity, education, income, exercise, nativity, and smoking.
